# Development of youth friendly family medicine services in Bosnia and Herzegovina: protocol for a cluster randomized controlled trial

**DOI:** 10.1186/2193-1801-3-319

**Published:** 2014-06-26

**Authors:** Dagmar M Haller, Françoise Narring, Patty Chondros, Daliborka Pejic, Ana Sredic, Senad Huseinagic, Nicolas Perone, Lena A Sanci, Anne Meynard

**Affiliations:** Primary Care Unit, Faculty of Medicine, University of Geneva, Geneva, Switzerland; Department of community, primary care and emergency medicine, Geneva University Hospitals, 4 av Gabrielle-Perret-Gentil, 1211 Geneve 14, Switzerland; Department of Pediatrics, Geneva University Hospitals, Geneva, Switzerland; Department of General Practice, The University of Melbourne, Melbourne, Australia; Foundation fami, Doboj, Bosnia & Herzegovina; Public Health Institute of Zenica-Doboj canton, Zenica, Bosnia & Herzegovina

**Keywords:** Family practice, Adolescent health services, Youth-friendly services, Cluster randomised trial, Quality of healthcare

## Abstract

**Background:**

Young people face many barriers in accessing health services that are responsive to their needs. The World Health Organization has led a call to develop services that address these barriers, i.e. youth-friendly health services. Addressing the needs of young people is one of the priorities of *Foundation fami*, an organisation working in collaboration with the Swiss Federal Department of Development and Cooperation and Geneva University Hospitals to develop quality family medicine services in Bosnia and Herzegovina. This paper describes the design of a trial to assess the effectiveness of a multifaceted intervention involving family medicine teams (primary care doctors and nurses) to improve the youth-friendliness of family medicine services in Bosnia and Herzegovina.

**Methods/Design:**

This is a stratified cluster randomised trial with a repeated cross-sectional design involving 59 health services in 10 municipalities of the canton of Zenica in Bosnia and Herzegovina. Municipalities were the unit of randomisation: five municipalities were randomised to the intervention arm and five to a wait-list control arm. Family medicine teams in the intervention arm were invited to participate in an interactive training program about youth-friendly service principles and change processes within their service. The primary outcome was the youth-friendliness of the primary care service measured using the YFHS-WHO + questionnaire, a validated tool which young people aged 15 to 24 years complete following a family medicine consultation.

A total of 600 young people aged 15 to 24 years were invited to participate and complete the YFHS-WHO + questionnaire: 300 (30 per municipality) at baseline, and 300 at follow-up, three to five months after the training program.

**Discussion:**

The results of this trial should provide much awaited evidence about the development of youth-friendly primary care services and inform their further development both in Bosnia and Herzegovina and worldwide.

**Trial registration:**

Australian New Zealand Clinical Trials Registry_ ACTRN12610000142033

## Background

### Youth-friendly family medicine services

Much of the current disease burden in young people is related to health-compromising behaviors and psychosocial problems including mental disorders, tobacco, alcohol and other substance use, accidents and injuries, sexually transmitted diseases and unwanted pregnancies (Gore et al. 2011). The dramatic physical, emotional, cognitive and social transformations of adolescence have implications for health care that are unique to this age group. Young people are in particular need of developmentally appropriate services to address this largely preventable disease burden. Yet many young people do not receive professional help for the problems that affect them (Elster and Kuznets 1994; World Health Organization 2014).

Studies in high income countries have shown that most young people, including those who engage in health compromising behaviors, visit a family doctor at least once a year (McPherson 2005; Murdoch and Silva 1996; Elliott and Larson 2004; Jeannin et al. 2005; Haller et al. 2008). Although little data on adolescent access to services is available worldwide, similarly high proportions of young people probably visit primary care doctors in low and middle income countries in which a strong emphasis is placed on primary care (Patton et al. 2012). Family doctors are therefore ideally placed to identify and respond to the common psychosocial burdens of youth. They can also contribute to preventing health compromising behaviors which have an impact on the development of non-communicable diseases in later life. Despite the burden of disease attributable to mental and behavioral disorders, the majority of consultations to family doctors are for somatic health problems (respiratory, dermatological…) (Haller et al. 2007; Ozer et al. 2002). Though many young people would trust advice from their health provider on such themes as contraception, substance use and sexually transmitted infections, few receive counseling about health-related behaviors in the consultation (Klein and Matos Auerbach 2002; Duncan et al. 2012).

Two decades of research have provided us with knowledge of the barriers young people face in accessing health services for the problems that most affect them (Gleeson et al. 2002; Sanci et al. 2005a; Donovan et al. 1997; Ozer et al. 2002).

Thus, we now have a clear understanding of the services young people require. The World Health Organization (WHO) summarised this view under the concept “youth-friendly services”, which are services that are available, accessible, acceptable, appropriate and equitable for young people. World-wide, projects have emerged to incorporate these principles of youth-friendly services into primary care in order to more appropriately respond to the needs of young people within existing health services. Whereas many of these projects are very promising, few have been appropriately evaluated (Tylee et al. 2007). Thus we currently lack strong evidence that incorporating youth-friendly principles into services is effective.

### Foundation fami project for the development of youth-friendly primary care services in Bosnia and Herzegovina

Foundation fami, which is supported by the Swiss Agency for Cooperation and Development, contributes to rebuilding and reorganizing primary care in Bosnia and Herzegovina (BiH) (Foundation fami 2014). Following the war, BiH has been working to reform its health system moving it from a high-cost specialist approach to a primary health care setting offering comprehensive and cost-effective health-care. According to the BiH Strategic Health Care Reform plan, family medicine in BiH has to take on a central role as a primary health care provider, acting as a gatekeeper to specialised care for all adults and for adolescents. Family medicine is organised around the family medicine team, which consists of one family doctor and one or two primary care nurses. Family medicine services can include one or several family medicine teams, depending on the size of the community they serve. Patients are registered with a specific team and can only consult other primary care professionals in cases of emergency.

Foundation fami participates in this reform by providing support to BiH institutions to perform education, re-construct health structures and re-organise health services.

The first phases of Foundation fami’s work, developed in collaboration with Geneva University Hospitals’ experts, focused on training and implementation of family medicine in Bosnia and Herzegovina. A new emphasis was then placed on coordinating and integrating health and social services into a coherent, cost-effective provision of services to the community, and in particular to vulnerable populations. This involved a series of projects, including broader public health campaigns, community outreach and education, links between schools and family doctors, youth involvement etc. (Meynard et al. 2009). Young people (defined by WHO as between 10 and 24 years) are considered as a vulnerable group as they face major barriers in accessing health care. In addition to the psycho-social health-burdens of young people described above, post-war Eastern European countries face societal changes that have great influence on young people’s health: unemployment, post-traumatic stress and children of traumatized parents, growing epidemics of sexually transmitted diseases, rising levels of tobacco and other substance use (Currie et al. 2012). Taking these elements into consideration and building on WHO’s principles, Foundation fami, in collaboration with a team from Geneva University Hospitals, has chosen to develop an intervention to improve the youth-friendliness of family medicine services in BiH.

This service development project provides the ideal opportunity for a well-designed evaluation.

#### Preliminary findings: validation of the YFHS-WHO + questionnaire

In 2008–2009 we conducted a study in six health services in BiH (four family medicine services and two emergency services) to validate an instrument for use in the present study: the YFHS-WHO + questionnaire (Haller et al. 2012). This validation study also acted as a pilot study to inform the methods and outcomes of this cluster randomised trial. It took place outside the canton of Zenica, the canton selected to conduct the cluster randomised trial.

The YFHS-WHO + questionnaire is a tool to measure the youth-friendliness of primary care services from a client (young people’s) perspective. It was adapted from a reproductive health services quality improvement questionnaire from WHO (World Health Organization 2009) and an Australian survey used to assess youth friendly primary care (Sanci and PARTY Research Team 2010). As the WHO tool was directed at sexual and reproductive health services, we used the Australian survey as a base to modify and add questions with the help of an international panel of experts. This ensured the tool was more suitable for use in the primary care context (family medicine). The tool was then translated into the language of BiH, pre-tested, refined and formally validated (Haller et al. 2012).

Construct validation involved 60 young people recruited in six different health services (10 per service). The health services (four family medicine services and two emergency services) had different levels of youth-friendliness according to expert evaluations. Young people completed the questionnaire after the consultation in a confidential interview with a member of the research team. Construct validity was supported by the fact that young people’s responses to the questionnaire led to higher scores for services which were specifically orientated towards young people according to expert evaluations. It was further supported by the finding that the proportion of young people consulting a service was higher in services with the highest YFHS-WHO + scores. Participants completed the same questionnaire seven to ten days after the initial completion and their responses were similar, indicating good test-retest stability. The initial tool comprised nearly 100 questions but item response analysis on subscales could be used to reduce the number of items in the questionnaire. The resulting YFHS-WHO + questionnaire is a 49-item tool with 8 subscales: access A (range of services) and B (structural aspects), parental support, equity, respect, privacy, no judgment, quality (Haller et al. 2012). The total score and the score on each subscale have a range between 0% and 100% where higher scores indicate higher levels of youth friendliness.

#### Aim and hypotheses

The aim of the present study was to assess the effectiveness of an intervention to improve the youth-friendliness of family medicine services.

Based on the results of a pilot study conducted in the context of the validation of the YFHS-WHO + questionnaire, our main hypothesis was that:

1. Health services exposed to the intervention will present more youth-friendly characteristics compared to services providing usual care, with an absolute minimal difference in mean scores on the YFHS-WHO + of 20% (range of possible scores 0-100%; assuming a standard deviation of 25).

Additional hypotheses were that:

2. Health services exposed to the intervention will fulfil a higher number of youth–friendly standards as assessed by members of the research team visiting the facility compared to usual care.
3. Health services exposed to the intervention will have a 10% higher proportion of young people between the ages of 10 and 24 years attending compared to the services in the usual care arm.

## Methods/Design

This was a cluster randomised controlled trial with municipalities as the unit of randomisation and two cross-sectional surveys of young people at baseline and three to five months post intervention. The intervention was delivered at the level of family medicine services within each municipality. We chose to randomise municipalities instead of the family medicine services to minimise the risk of contamination between family medicine teams located in close proximity to each other. Teams belonging to the same municipality usually attend similar continuous medical education (CME) activities and are likely to interact frequently, increasing the chance of sharing information and learning from each other.

### Participants: recruitment and randomisation

The study involved family medicine services in all 10 municipalities of the canton of Zenica. Approval was sought from the Head of family medicine services in each municipality, who then invited the family medicine teams to participate. The teams were not offered any incentives other than the opportunity to participate in a continuous medical education (CME) program and thus to obtain CME credits. A computer generated randomisation list was generated by an independent statistician based in Geneva, Switzerland. Due to the small number of municipalities to be randomised, randomisation was stratified by size of municipalities (5 small municipalities (<6 participating teams/municipality); 5 larger municipalities (≥6 participating teams/municipality)), to balance the number of family medicine services between the study arms. In smaller municipalities, all family medicine teams were invited to participate. In larger municipalities, a maximum of 10 teams were randomly selected to participate (see sample size calculations).

In each service, consecutive young people between the ages of 15 and 24 years consulting for any motive were approached and invited to be part of the study. There were no exclusion criteria for family medicine services (any family medicine team in the canton of Zenica could participate). Patient exclusion criteria were: acute illness or injury requiring immediate attention of the physician, severe mental disorder such as psychosis or suicidal thoughts requiring treatment in a specialised setting, intellectual disability and inability to understand questions in the language of BiH.

### Data collection

Following consent by the family medicine team, measures were collected at baseline, and between three to five months after the implementation of the intervention. The youth-friendliness of participating family medicine services were assessed with a different sample of young people consulting the services (repeated cross-sectional samples) using the same procedure for recruitment of young people and data collection at baseline and follow-up.

Trained research assistants (nursing students) made contact with the young people within the family medicine service to obtain their consent and arranged a meeting for an interview in which they completed the questionnaire either in the family medicine service or in a more neutral location, within 48 hours of attending the practice. Meeting outside the service allowed the young people to be more freely critical of the service if they wished to. The research assistant provided potential participants with an information sheet and a consent form to sign before young people completed the questionnaire. The questionnaire consisted of demographics questions, such as age, sex, school or professional situation and the YFHS-WHO + tool. As mentioned above, the YFHS-WHO + questionnaire is a validated tool specifically developed for this study (Haller et al. 2012). The questionnaire was completed anonymously (there was only a code number on the questionnaire and not the patients’ names). The young people did not receive any incentives for participation in the study.

Though the aim of the intervention was to adapt family medicine services for young people between the ages of 10 and 24 years, only young people from the age of 15 years were asked to complete the YFHS-WHO + questionnaire. The age restriction was based on the following consideration: young people have repeatedly identified confidentiality as a key priority when accessing health care (Sanci et al. 2005b). In order to respect this confidentiality, it was important that young people recruited for the study could consent to participate without asking their parents (Haller et al. 2005). As previous experience in several countries has shown, young people from the age of 15 years are capable of consenting to participation in a low risk project like this one on their own (Sanci et al. 2004). In addition, the YFHS-WHO + questionnaire used in the study was validated for young people from the age of 15 years to 24 years (Haller et al. 2012).

All family medicine services were subject, at baseline and follow-up, to a qualitative evaluation of youth-friendly standards by a member of the research team, using a standardised grid (based on the WHO tool) and interviews of the health professionals working in the family medicine service (World Health Organization 2009). Summary data on the proportion of young people between the ages of 10 and 24 years attending the services in the past 3 months were also collected, at baseline and at follow-up.

### Intervention

The family medicine teams in the intervention arm were invited to attend a total of three days of training on youth friendly services. The training was designed and facilitated by two of the principal investigators, one from Switzerland (AM) and one from BiH (DP). The cultural appropriateness of the content was thus ensured. Spontaneous exchanges in the local language were also facilitated. A professional translator was present during the entire training to translate English interventions and answers or discussions with participants. The intervention was based on a 2 day educational course developed by AM used to educate family medicine teams on youth friendly services in another province of Bosnia and Herzegovina in 2008. The course was well accepted by participants and used as a pilot study to adapt material and content for this study.

Two separate one day modules were delivered three months apart (Table 1). The educational principle of experiential learning was used during training via the modalities of group work, role plays and observation of videos (Kolb 1984). In addition there were lectures and learning activities helping to bridge theory, local context and participant’s personal experiences, such as case discussions in relation to confidentiality issues. In the 3 months between the delivery of the two modules, participating clinicians completed personal assignments as outlined in Table 1, for which they received ongoing support of facilitators. The clinicians presented and discussed their personal assignments during the second module. Participants received a full portfolio with the content of the courses and all the projects presented by participants in the second module. Participants completed a satisfaction survey at the end of the training, a prerequisite for the training to be eligible for provision of CME credits. Completion of both modules led to a certificate and CME credits.

**Table 1:** **Main topics covered in the training**

**Module 1 (one day)**	**Morning:** A developmental definition of adolescence and epidemiological data With the following questions in mind (from a family medicine point of view)
	1. *What should we do differently for adolescents?*
	2. *Why invest in adolescent health?*
	3. *What is the contribution of family medicine teams in our regions?*
	**Afternoon:** What do I do when I meet an adolescent in my practice?
	Confidential care, principles of communication and the HEADSSS mnemonic*
	**→ At the end of the module**: Evaluation and Presentation and distribution of assignment tasks

*HEADSSS is a mnemonic to guide psychosocial interviews with adolescents (Home, Education/Eating, Activities, Drugs, Sexuality, Safety, Suicide) (Goldenring and Rosen 2004).

Family medicine teams in the control arm received the opportunity to participate in the training after the study (wait list control arm).

#### Allocation concealment and blinding

At baseline, the allocation of the municipalities to the study arms was concealed until after the young people were recruited and data collected. DH then assigned the municipalities to the intervention or control arms. Once the municipalities had been assigned to the study arms, it was not possible to blind the family medicine teams because of the nature of the intervention. However, research assistants involved in the recruitment of young people and collection of the outcome data were blinded to the study arm status of the family medicine services. Young people were also blinded to the study arm status.

### Outcome measures

The primary outcome measure was the score on the adolescent client YFHS-WHO + questionnaire. Secondary outcomes were the number of youth friendly standards attained by the family medicine service (as assessed on the standardised grid), and the proportion of young people between the ages of 10 and 24 years attending the service.

### Ethical approval and study registration

As there was no ethical committee to approve the project in the canton of Zenica in which the project was to be undertaken, the protocol was submitted to the ethical committee of Geneva University Hospitals, Switzerland. This ethical committee was chosen because a) several lead investigators for the study are from this institution, b) Geneva University Hospitals have been involved in helping rebuild health services in BiH after the war and therefore the context and structures are known to members of this institution. This committee gave formal approval for the project on the basis of documents in English. The patient information and consent documents were then translated in the language of BiH and the accuracy of the translation was checked by bilingual members of the team.

### Sample size estimation

Fifty-nine family medicine teams across the ten municipalities of the canton of Zenica participated in the study. This number of family medicine teams was fixed by the public health authority supporting the trial. The number of teams available for the trial was based on achieving a balance between including all family medicine teams in the canton wishing to participate in the project (a unique opportunity for continuous medical education) and the requirement for some teams in some municipalities to remain available for patients during the training periods (depending on the availability of other services such as emergency services in the area).

**Figure 1:**
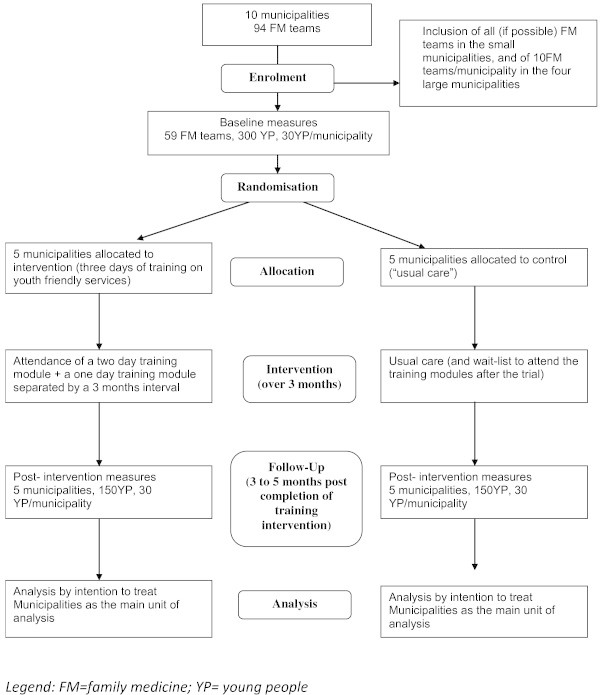
**Flow diagram of the research plan.** *Legend: FM = family medicine; YP = young people.*

In total, 300 young people across the 10 municipalities were required at baseline and another 300 post-intervention. For an individually randomised trial, a total of 50 young people were required in both study arms to detect an absolute difference of 20% in mean scores on the YFHS-WHO + questionnaire between the study arms (standard deviation = 25%, alpha = 5%, power = 80%, 2-sided test). To allow for clustering by municipality, the sample size for the individually randomised trial was inflated by a factor of 4.6 (design effect), assuming that the intra-cluster correlation (ICC) was 0.163 for 10 clusters, based on a study using a similar tool (Potiriadis et al. 2008). Hence, based on the assumptions we would require 23 individuals per municipality (that is a total of 230 young people). We increased the total number of individuals per municipality to 30, which allows for the degrees of freedom correction based on 8 clusters assuming a slightly lower intra-cluster correlation of 0.13 (and inflation factor of 4.8), because the number of clusters was small. In municipalities with 10 family medicine teams included in the study (eg Kakanj, city of Zenica), a minimum of three individuals per family medicine service were needed to complete the questionnaire, whereas in smaller municipalities, up to 15 individuals per service were required as presented in Table 2 and Figure 1.

**Table 2:** **Sample size and patient (young people aged 15 to 24 years old) recruitment plan for each municipality**

Name of municipality	Number of family medicine teams in this municipality	Number of teams expected to participate in the study	Number of individuals per FM team needed ***(rounded up)***	Minimum number of individuals per municipality
**Small municipalities**				
Breza	4	4	8	30
Olovo	6	4	8	30
Usora	2	2	15	30
Vores	4	4	8	30
Zepce	3	3	10	30
**Large municipalities**				
Maglaj	6	6	5	30
Kakanj	13	10	3	30
Visoko	14	10	3	30
Zanidovici	10	6	5	30
Zenica	32	10	3	30
			**Total youth required**	**300**

#### Analysis strategy

Stata software will be used for the analysis. Family medicine services and young people’s characteristics will be summarised using frequencies and percentages for categorical data and means and standard deviations for continuous data. To assess for chance imbalance in the sample, the characteristics of the family medicine teams and outcomes measured at baseline will be compared between the intervention and control arms. Analysis will be by intention to treat. Summary measures of the outcomes will be calculated for each municipality; namely, means for the total YFHS-WHO + score and its eight sub-scales and proportions for binary outcomes. Linear regression of the cluster-level summary outcomes on the study arm status will be used to estimate the intervention effect. The intervention effect will be reported as difference in means between the study arms for continuous outcomes and difference in proportions for binary outcomes, with respective 95% confidence intervals and p-values. Multiple linear regression will be used to adjust estimates of the intervention effect for the outcomes measured at baseline, summarised as means or proportions for each municipality, and the size of municipality (that is, <6 family medicine teams and ≥6 family medicine teams).

### Potential contribution of this study to the field

The results will be published in medical journals and presented at national and international medical conferences. If the intervention is effective, the next phase will be implementation through extending the training of family medicine teams at a national level, and extending the findings to an international level. The results of the trial and the experience acquired in the project will also act as a step in further promoting the development of Youth Friendly Health Services as recommended by the World Health Organisation (WHO 2002).

## Abbreviations

BiH: Bosnia and Herzegovina
CME: Continuous medical education
FM: Family medicine
WHO: World Health Organization
YP: Young people.
